# Return to Sport and Performance After Treatment of Osteochondral Lesions of the Ankle in Elite Athletes: A Retrospective Study

**DOI:** 10.1177/23259671261422247

**Published:** 2026-03-31

**Authors:** Carlijn S. ter Laak Bolk, Julian J. Hollander, Elze J. Geurts, Kishan R. Ramsodit, Quinten G.H. Rikken, Jari Dahmen, Sjoerd A.S. Stufkens, Gino M.M.J. Kerkhoffs

**Affiliations:** * Department of Orthopedic Surgery and Sports Medicine, Amsterdam UMC, University of Amsterdam, Amsterdam, the Netherlands; † Amsterdam Movement Sciences, Program in Sports and Musculoskeletal Health, Amsterdam, the Netherlands; ‡ Academic Center for Evidence-Based Sports Medicine (ACES), Amsterdam, the Netherlands; § Amsterdam Collaboration for Health and Safety in Sports (ACHSS), International Olympic Committee Research Center, Amsterdam, the Netherlands; Investigation performed at Amsterdam University Medical Centers, Amsterdam Medical Center, University of Amsterdam, Amsterdam, the Netherlands

**Keywords:** ankle, elite athletes, osteochondral lesions, return to sports, talus, tibia

## Abstract

**Background:**

Osteochondral lesions of the ankle (OLA) often occur in athletes, necessitating a focus on sport outcomes such as return to sports (RTS) and return to performance (RTP). Only a few studies have evaluated sports outcomes after treatment of an osteochondral lesion in the athletic population, let alone in an elite athlete population.

**Purpose:**

To assess RTS and RTP rates and times after treatment of OLA in elite athletes.

**Study Design:**

Case series; Level of evidence, 4.

**Methods:**

All elite athletes treated for an OLA were selected from a cross-sectional database consisting of consecutive patients with cartilage injuries of the ankle until August 2022. Outcomes included RTS outcomes, patient-reported outcomes, and a quantitative assessment regarding mental health.

**Results:**

A total of 25 patients, with a median age of 22 years (interquartile range [IQR], 19-25 years), were included, with a median follow-up time of 48 months (IQR, 35-71 months). Six patients (24%) underwent nonoperative treatment, and 19 patients (76%) underwent operative treatment. Nonoperative treatment yielded RTS rates of 83% at any level, 67% at the preinjury level, and 67% for RTP. For operative treatment, these rates were 100%, 74%, and 63%, respectively. The median time to return to preinjury level was 7.5 months overall.

**Conclusion:**

The different treatment options for OLAs allowed for adequate RTS and RTP outcomes. Overall, an RTS rate of 96% at any level, a return to the preinjury level of 72%, and an RTP of 64% were found. These sport-specific outcomes can inform athletes about their expected outcomes after different treatments for OLAs.

Osteochondral lesions of the ankle (OLA) are common in elite athletes, who experience high ankle injury rates, limiting their ability to perform at competitive levels.^13,21,24,34^ Most patients with an OLA present 6 to 12 months after initial trauma with deep ankle pain aggravated during or after weightbearing activities, while other patients complain of chronic ankle pain.^24,26^ These symptoms can limit the ability of athletes to perform at their desired level.^8^

Treatment is individualized based on patient factors, lesion characteristics, concomitant injuries, and ankle deformities.^24^ Treatment options range from nonoperative (eg, activity restriction, physical therapy, immobilization, and nonsteroidal anti-inflammatory drugs) to operative (eg, bone marrow stimulation [BMS], internal fixation, and autografting), tailored to lesion severity and athlete needs. Only a few studies have evaluated the sports outcomes after treatment of OLAs in the athletic population.^9,18,22,27,28,31^ However, only 2 studies report on 26 elite athletes and only describe the results of arthroscopic debridement.^27,28^ Thus, no literature is available on the treatment of OLAs in elite athletes, with existing literature limited to arthroscopic debridement in small cohorts.

Emphasis on sport outcomes such as return to sport (RTS) and return to performance (RTP) is critical for elite athletes, given the high physical and professional demands of their careers, unlike amateur athletes who may prioritize recreational participation. This is because most athletes remain highly motivated and disciplined to return to their sport and compete at the highest level available.

Therefore, this study aimed to assess the RTS and RTP rates of OLAs in elite athletes, treated either operatively or nonoperatively. Secondarily, other RTS outcomes were assessed, along with the RTS times for all outcomes.

## Methods

### Study Design

The present cross-sectional study was approved by the Medical Ethical Committee of the Amsterdam University Medical Centers (UMC) (W14_237#14.17.0288). The study was also performed in accordance with the Declaration of Helsinki. Patients were included from a cross-sectional database spanning January 1989 to August 2022 and were treated at the Amsterdam UMC, a tertiary academic referral center, accredited as an official international expert center for cartilage damage of the ankle and foot and as an officially recognized International Olympic Committee Research Center of Excellence.

### Patient Selection

To identify all patients with an OLA, the database mentioned above was used. The electronic patient records were then retrospectively screened to identify potentially eligible patients using the inclusion and exclusion criteria (Table 1). All patients were treated by 2 orthopaedic surgeons (S.A.S.S. and G.M.M.J.K.) between June 2008 and August 2022. For this study, an elite athlete was defined as an individual who, at the time of injury, competed at a minimum of national level (ie, the highest and second-highest levels) in their respective sport (individual or team).^19^

**Table 1:** Inclusion and Exclusion Criteria*^*a*^*

Inclusion Criteria	Exclusion Criteria
- Elite athlete with a symptomatic osteochondral lesion of the talus, as assessed by physical examination and radiological assessment (S.A.S. and/or G.K.)	- Concomitant injuries (eg, acute fractures, advanced osteoarthritis (≥3 grade according to Takakura et al^32^), infectious pathology, malignancies)
- All treatment options (eg, nonoperative or operative)	- Asymptomatic OLA
- Follow-up of >6 months	

a OLA, Osteochondral lesions of the ankle.

### Data Collection

After screening, eligible patients were contacted by phone or email to obtain consent for participation in this study. All included patients were sent an online questionnaire via the CASTOR portal to collect information regarding patient-reported outcome measures (PROMs). Baseline characteristics of the patients, sports, injuries, lesions, and treatments (such as age, sex, body mass index, type of sport, level of sport, lesion size, lesion location, lesion morphology, and type of treatment) were collected from the electronic patient records. Furthermore, outcome scores for sports, RTS (yes/no and timing), pre- and postoperative level of sports, and type of sports were collected via an in-depth telephone interview. All questionnaires were reviewed with patients during phone interviews to ensure comprehension.

### Treatment Options

Patients were treated both operatively and nonoperatively. The treatment decision was made in a shared decision-making process, based on the patient characteristics and lesion characteristics, as well as the patient and clinician preferences. This decision-making process has been described in a previous publication.^24^

Nonoperative treatment consists of one of the following pillars: adjustment of activities/physical therapy, insoles, bracing, weightbearing restrictions, and/or injectables.^2,6,24^

If operative treatment was indicated, several techniques were used. These included arthroscopic BMS,^4,23^ open or arthroscopic fixation by means of the Lift-Drill-Fill-Fix (LDFF) technique,^15^ or osteoperiosteal autografting by means of the talar osteoperiostic grafting from the iliac crest (TOPIC) technique.^14^ The postoperative rehabilitation protocol was implemented as described in these technique papers.

### Outcomes

#### Sport Outcomes

The primary outcome of this study is the RTP rate. Other outcomes regarding sports collected in this study were return to any level of sport and return to the preinjury level. Lastly, the RTS (in months) for return to any level of sport, return to the preinjury level, and RTP were collected.

RTS and its subcategories were defined according to a modification of Ardern et al.^1^ The levels of RTS and their definitions used in this study are shown in Table 2.

**Table 2:** Levels and Definitions of RTS*^*a*^*

Level of RTS	Definition of Level
RTALS	Return to any level of sport regardless of preinjury level, with the patient being active in sports (ie, rehabilitation, modified/restricted training), but not ready to be active at the preinjury level
RTSLS	The return to the same level and type of sport as before injury in training
RTP	Performing at the level of sports achieved without the injury, or even at a higher level, compared with the preinjury situation

a RTALS, return to any level of sport; RTP, return to performance; RTS, return to sports; RTSLS, return to preinjury level of sport.

Information regarding sports was collected via an in-depth telephonic interview by one of the authors (C.tLB.). All patients were asked whether they participated in sports, the type of sport, and the level of sport participation (elite, competitive, or recreational) before their injury. Patients were asked about their ability to RTS after treatment (yes/no). For those who were able to RTS, information was collected on the levels of RTS as defined in this study (Table 2), along with the time required to achieve each level. Furthermore, patients were asked at what level of sport participation they could return. In cases where patients were unable to resume sports after treatment, the reasons for this inability were asked. Finally, at the latest follow-up, patients were asked whether they still participated in sports, which type of sport they participated in, and at what level.

#### Clinical Outcomes

The PROMs were collected at follow-up. The clinical outcomes included the Numeric Rating Scale (NRS) of pain during rest and during weightbearing,^33^ the Short Form-12 (SF-12), and the Foot and Ankle Outcome Score (FAOS).^20,29^ From the SF-12, both the Physical Component Summary (PCS) score and Mental Component Summary score were calculated.

### Mental Health

An online/electronic quantitative retrospective assessment of the mental health of all patients was conducted using modified, validated questionnaires for mental health symptoms, as reported by Cannon et al.^3^ All patients were asked whether they experienced symptoms related to mental health during injury, treatment, and the rehabilitation period. Symptoms related to mental health problems were measured using the following domains: anxiety, depression, sleep disturbance, and alcohol misuse (yes/no questions).

Additionally, the patients’ current mental health status was assessed using validated questionnaires. Anxiety was assessed^30^ using the General Anxiety Disorder-7. Depression was measured^16^ using the Patient Health Questionnaire 9. The level of alcohol misuse was measured using the Alcohol Use Disorders Identification Test–Consumption.^5^

### Complications and Reoperations

Any complications related to the treatment received, revision surgeries, or reoperations of the ankle or foot after operative treatment were analyzed. The definition of a complication has been defined in a previous study.^11^ Revision surgery was defined as any surgical procedure involving treatment of the cartilage or bone of the ankle joint after original intervention. The relevant data were retrieved from the electronic patient records.

### Radiological Assessment

An assessment of the pretreatment computed tomography (CT) scan was performed by 2 researchers (C.tLB. and J.D.) to assess pretreatment lesion characteristics. Lesion characteristics assessed were lesion size, lesion morphology, and lesion location. The lesion size was determined (in mm) from anterior-posterior (AP), medial-lateral (ML), and depth. Lesion morphology was classified as cystic, fragmentary, or crater, as defined by Rikken et al.^25^ Lesions were localized using a 9-grid scheme.^7^ In case of disagreement after a discussion, a third author (G.K.) made the final decision.

### Statistical Analysis

Patient characteristics were summarized using descriptive statistics. Categorical data were presented as a frequency. Continuous outcomes were visually assessed for normal distribution. When a normal distribution was observed, the data were presented as means with standard deviations. For non-normally distributed data, continuous outcomes were reported as medians with corresponding interquartile ranges (IQRs).

The primary outcome (RTP) and other RTS outcomes were calculated for the total population. Current mental health was reported as the occurrence of mental health symptoms (MHS) and was calculated as a proportion of the number of participants with MHS compared with the total population. For this, the modified Wald method was used to obtain 95% CI.^35^

Statistical data analysis was performed using SPSS, Version 28 (IBM).

## Results

### Patient Characteristics

**Figure 1.**
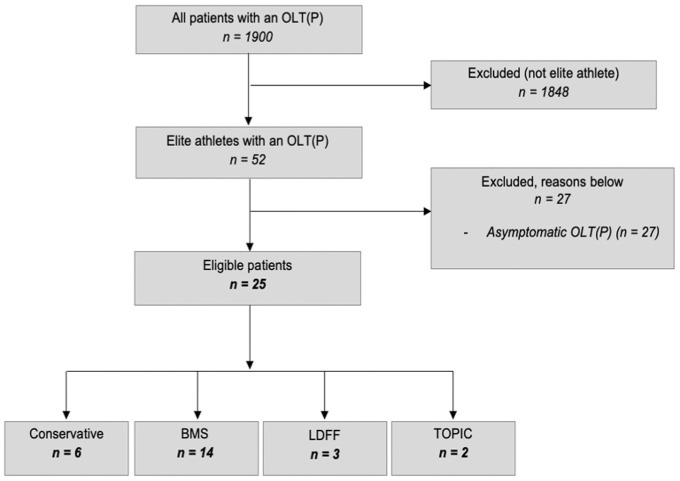
Flowchart of patient selection process.

From the database, 52 elite athletes were identified. Based on the inclusion and exclusion criteria, 25 patients were approached for this study; 100% responded and participated. The patient selection process is shown in Figure 1. The median age was 22 years (IQR, 19-25 years), and the median follow-up time was 48 months (IQR, 35-71 months). Six patients (24%) underwent nonoperative treatment. For operative treatment, 19 patients (76%) were identified, with BMS as the most used operative technique. A full overview of baseline characteristics is presented in Table 3.

**Table 3:** Overview of Baseline Characteristics*^*a*^*

Treatment Group	Patients, n	Age, Years	Sex	BMI, kg/m^2^	Laterality, Left, n	Follow-up, Months,	Concomitant Surgeries, n	Preinjury Type of Sports, n	Ankle Activity Score
Nonop	6	17 (15-27)-	Male: 2; female: 4	23 (21-26)	Left: 2; right: 4	43 (14-52)	0	Basketball (1), cricket (1), volleyball (1), acrobatic gymnastics (1), soccer (1), and field hockey (1)	9 ( 8.5-10)
Op	19	22 (20-24)	Male: 13; female: 6	24 (22-26)	Left: 10; right:	48 (30-80)	12		9 (6-10)
BMS	14	23 (22-26)	Male: 11; female: 3	24 (22-25)	Left: 8; right: 6	47 (17-81)	11	Soccer (3), dancing (1), gymnastics (2), free running (1), rugby (1), skateboarding (1), tennis (1), ballet dancer (1), karate (1), basketball (1), and padel (1)	9.5 (7.5- 10)
LDFF	3	22 (16-22)	Male: 2; female: 1	22 (22-25)	Left: 2; right: 1	46 (30-62)	1	Rhythmic gymnastics (1), speed skating (long track, 1), and baseball (1)	7 (5-9)
TOPIC	2	Mean, 21 (range, 19- 23)	Male: 0; female: 2	Mean, 26 (range, 25 -26)	Left: 0 right: 2	Mean, 95 (range, 54-14)	0	Dancing (1) and equestrian (1)	Mean, 5 (range, 3-6)

a Data are presented as median (IQR). BMI, body mass index; BMS, bone marrow stimulation; IQR, interquartile range; LDFF, Lift-Drill-Fill-Fix; Nonop, nonoperative; Op, operative; TOPIC, talar osteoperiosteal grafting from the iliac crest.

### Radiological Assessment

**Figure 2.**
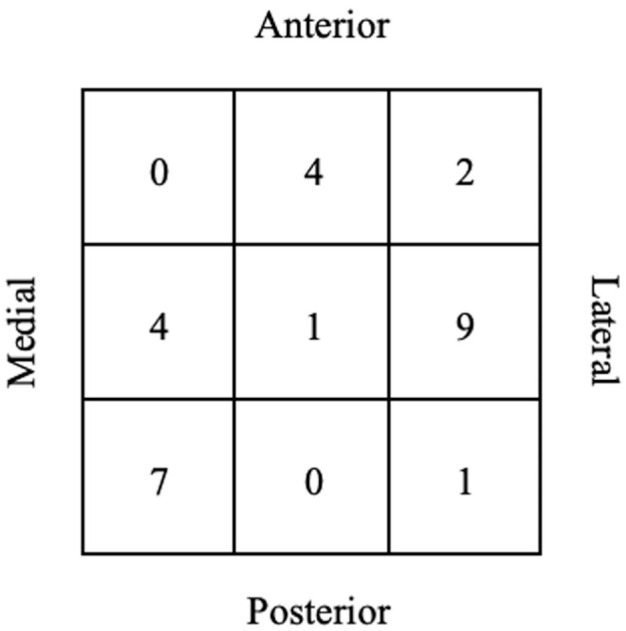
Overview of lesion location of OLAs.

In total, 21 patients (84%) had an osteochondral lesion of the talus (OLT), 4 patients (16%) had an osteochondral lesion of the tibial plafond (OLTP), and 2 patients (8%) had both an OLT and an OLTP. Figure 2 shows the distribution of the lesion location according to the 9-grid scheme. The intraclass correlation coefficient value for interobserver agreement was 0.98 for AP diameter, 0.97 for ML diameter, and 0.91 for depth. These outcomes can be considered excellent. Table 4 provides an overview of radiological outcomes.

**Table 4:** Radiological Characteristics*^*a*^*

Morphology	
- Cystic lesion	10 (36)
- Fragmentary lesion	6 (21)
- Crater lesion	12 (43)
OLTs	
AP diameter, mm	13 (6-15)
ML diameter, mm	6.2 (4.7-9.2)
Depth, mm	5 (3.1-5.3)
Mean lesion size, mm^2^	0.7 ± 0.4 ( 0.1-1.3)
Mean lesion volume, mm^3^	0.6 ± 0.4 ( 0.1-1.6)
OLTPs	
AP diameter, mm	11 (6.9-18)
ML diameter, mm	9.2 (8.6-14)
Depth, mm	7.1 (3.8-9.6)
Mean lesion size, mm^2^	1.2 ± 1.2 (0.3-3.4)
Mean lesion volume, mm^3^	1.3 ± 1.8 ( 0.1-5)

a Data are presented as n (%), median (IQR), or mean (range). AP, anteroposterior; IQR, interquartile range; ML, mediolateral; OLTPs, osteochondral lesion of the tibial plafond; OLTs, osteochondral lesion of the talus.

### Sport Outcomes

Overall, 96% (24/25) of patients returned to sport at any level, 72% (18/25) at the preinjury level, and 64% (16/25) at the performance level. In the nonoperative group, 83% (5/6) returned to any level, 67% (4/6) to the preinjury and performance level, compared with 100% (19/19), 74% (14/19), and 63% (12/19) in the operative group (Table 5). The median time to performance level was 10 months. There were various reasons why, for some patients, RTS was not possible: 4 patients reported that they suffered from persistent pain in the ankle, 3 patients were unable to perform certain movements with the ankle necessary for their sport, and 2 patients reported taking on other interests in life as a reason for no RTS.

**Table 5:** RTS Outcomes*^*a*^*

Treatment	RTS Rate	Median RTS Time, Months
All treatment options, n = 25		
- Return to any level	24/25	3 (2-5)
- Return to preinjury level	18/25	7.5 (4-12)
- Return to performance	16/25	10 (4.9-13)
Nonoperative treatment, n = 6		
- Return to any level	5/6	3 (1.3-6.8)
- Return to preinjury level	4/6	3.5 (2.3-10)
- Return to performance	4/6	4.3 (3.3-10)
Operative treatment, n = 19		
- Return to any level	19/19	3 (2-5)
- Return to preinjury level	14/19	8.5 (5.5-12)
- Return to performance	12/19	11 (8-14)
BMS, n = 14		
- Return to any level	14/14	4 (2.8-5.1)
- Return to preinjury level	10/14	9 (4-12)
- Return to performance	10/14	11 (7.5-13)
LDFF, n = 3		
- Return to any level	3/3	2 (1.5-3)
- Return to preinjury level	2/3	12 ( 6-18)
- Return to performance	1/3	18
TOPIC, n = 2		
- Return to any level	2/2	4.5 (3-6)
- Return to preinjury level	2/2	6.5 (6-7)
- Return to performance	1/2	8

a Data are presented as median (IQR) or median (range). BMS, bone marrow stimulation; LDFF, Lift-Drill-Fill-Fix; RTS, return to sports; TOPIC, talar osteoperiosteal grafting from the iliac crest.

### Patient-Reported Outcomes

The median NRS in rest was 1 and 2 during running at the latest follow-up. The overall FAOS score also showed good results across the various subscales, especially in the Pain (89) and Activities of Daily Living (99) subscales. An overview of the clinical outcomes is presented in Table 6.

**Table 6:** Clinical Outcome Scores*^*a*^*

	NRS				FAOS					SF-12	
	Rest	Walking	Stairclimbing	Running	Symptoms	Pain	ADL	Sport	QoL	PCS	MCS
Overall	1 (0-5)	2 (1-3.5)	2.5 (1-4.5)	2 (1-4.3)	71 (48-89)	89 (86-96)	99 (92-100)	80 (63-90)	75 (47-88)	55 (53-57)	56 (56-58)
Nonop	0 (0)	1 (0-2)	0 (0)	2 (1.5-3)	71 (71-93)	92 (89-99)	100 (98-100)	80 (75-85)	75 (57-88)	57 (54-57)	56 (56-58)
Op	0 (0)	0 (0-1.8)	0.5 (0-2.8)	1 (0-3.8)	59 (44-89)	89 (84-94)	97 (87-100)	78 (53-94)	78 (44-88)	55 (53-88)	55 (38-57)

a Data are presented as median (IQR). ADL, activities of daily living; FAOS, Foot and Ankle Outcome Score; IQR, interquartile range; MCS, Mental Component Summary; Nonop, nonoperative treatment; NRS, Numeric Rating Scale; Op, operative treatment; PCS, Physical Component Summary; SF-12, 12-Item Short Form Health Survey; QoL, quality of life.

### Mental Health

A total of 21 patients reported on mental health. Anxiety was the most reported symptom, peaking at 52% during rehabilitation. Depression was most common during rehabilitation (38%). Currently, most patients report minimal or mild anxiety (86%) and depression (81%). Table 7 presents all prevalence rates of mental health symptoms.

**Table 7:** Prevalence of Mental Health Symptoms, n = 21*^*a*^*

During injury, %	
- Anxiety	48
- Depression	24
- Sleep disturbance	29
- Alcohol misuse	0
During the treatment period, %	
- Anxiety	48
- Depression	24
- Sleep disturbance	29
- Alcohol misuse	0
During rehabilitation, %	
- Anxiety	52
- Depression	38
- Sleep disturbance	24
- Alcohol misuse	0
Current mental health	
Anxiety, GAD-7	7 (0-3)
- Minimal, 0-4, n	19
- Mild, 5-9, n	None
- Moderate, 10-14, n	1
- Severe, 15-21, n	1
Depression, PHQ-9	1 (0-3.5)
- Nonminimal, 0-4, n	17
- Mild, 5-9, n	2
- Moderate, 10-14, n	2
- Moderately severe, 15-19, n	None
- Severe, 20-27, n	None
Alcohol misuse, AUDIT-C	2 (1-4)
- Low risk, 0-4, n	19
- Increasing risk, 5-7, n	2
- Higher risk, 8-10, n	None
- Possible dependence, 11-12, n	None

a Data are presented as median (IQR), unless otherwise indicated. AUDIT-C, Alcohol Use Disorders Identification Test–Consumption; GAD-7, Generalized Anxiety Disorder–7; IQR, interquartile range; PHQ-9, Patient Health Questionnaire–9.

### Complications And Reoperations

There were no complications during surgery. Two weeks after surgery, 1 patient suffered from a superficial wound infection in the BMS group, which was adequately treated in a hospital at the place of residence of this patient. This did not affect the patient's ability to RTS. In total, 2 of the 5 patients undergoing open surgery (40%) required hardware removal at 12 and 36 months after initial surgery, respectively.

## Discussion

This study evaluated the RTS rates and durations across multiple treatment options for ankle osteochondral lesions in elite athletes. The primary finding is an overall RTP rate of 64% among elite athletes after treatment for OLAs, with a median return time of 10 months. However, there is a notable decline in RTS rates at higher levels of RTS.

When considering RTS rates across different treatment options, a consistent trend emerges. While the nonoperatively treated group shows a return-to-sport rate of 83%, this rate decreases to 66% for return to preinjury levels and RTP. Conversely, the operative group demonstrates a 100% return to any level of sport, yet the percentage of return to preinjury levels (74%) and RTP (63%) decreases. Several factors contribute to the inability of some patients to return to their preinjury or performance levels of sport—including persistent ankle pain, limitations in performing sport-specific movements involving the ankle, or shifts in focus toward alternative life interests. However, rehabilitation demands vary by sport; low-impact activities such as swimming may allow faster recovery than high-impact sports such as running, consistent with general rehabilitation principles.^12^ Because of the small sample size and diversity of sports (Table 3), subgroup analysis by sport type was not feasible, although outcomes varied across individual cases.

Steman et al^31^ found adequate RTS rates after surgical treatment of OLTs. The return to any level of sport was 88%, which is lower compared with the present study. In their study, the RTS rate at preinjury levels dropped to 79%—a significant decrease. However, the level of sport at the time of injury was not reported; therefore, comparisons with a population consisting only of elite athletes may not be appropriate. A study by Sheu and Ferkel^28^ found that all 10 National Basketball Association players included in their study were able to RTS after arthroscopic debridement of their OLTs in the same or the following season at the preoperative level. Seijas et al^27^ reported a return to preinjury sport level of 94% in a group of 16 elite soccer players after arthroscopic debridement and BMS for OLTs at a mean follow-up of 3.6 years. In a systematic review by Hurley et al,^12^ an RTS rate of 86.6% was observed after a mean of 4.5 months after BMS among 248 athletes. The athletes’ level was not specified in the study. One potential explanation for the higher percentages observed in other studies may stem from their smaller sample sizes. For instance, 2 of the studies focused exclusively on a single sport, potentially limiting the diversity of athletes included. In contrast, this study encompasses a broad range of sports practiced by elite athletes, offering a more comprehensive understanding of sport-specific outcomes across various athletic disciplines.

In a prospective study by Lambers et al^18^ on 60 athletes, the NRS and FAOS were reported at final follow-up at approximately 6 years. The NRS at rest was 1 and 4.3 during activity at final follow-up. In this study, these outcomes were 1 and 2, respectively. Additionally, the present study found higher scores across all FAOS subscales.

Another important role of the RTS drive in elite athletes is its impact on mental health. Over the years, attention to symptoms of mental health problems has increased among professional athletes.^17^ Elite athletes in this study reported notable mental health symptoms of anxiety (48%-52%) and depression (24%-38%) during injury, treatment, and rehabilitation. Unfortunately, only 21 of the 25 athletes in this study completed the mental health questionnaires, highlighting that this remains a difficult or potentially confronting topic for some to discuss. These findings align with previous research indicating that injuries can exacerbate mental health symptoms in elite athletes.^10^ However, the present study did not statistically assess their effect on RTS because of the small sample size. The prevalence of these symptoms underscores their relevance, and future research should investigate their influence on RTS outcomes to guide targeted interventions.

The results of this study must be interpreted in light of its strengths and weaknesses. A limitation of this study is the small sample size. Because only 25 patients were included, results should be interpreted with caution. It is, however, to the best of the authors’ knowledge, one of the largest studies available on RTS rates in elite athletes after OLA treatment. Another limitation is the heterogeneity of the study population, as the follow-up time and the different lesion measurements showed large ranges. The inclusion period may introduce time-lead bias due to advancements in surgical techniques and rehabilitation protocols, although all treatments followed standardized protocols at a specialized center. Moreover, not all patients had a posttreatment CT scan; therefore, no radiological assessment could be performed at the latest follow-up. Additionally, this study did not assess the Tegner scale, and the ankle axis was not measured. Lastly, the retrospective nature of the present study is a limitation, as it may introduce recall bias regarding the RTS outcomes.

Because managing the expectations of elite athletes is often a large part of the treatment process, outcomes regarding the possibility of RTS and return to the level of sport after treatment of OLAs are very important. The results of this study can be used in the expectation management process between the physician and patient, in this case, the elite athlete. Although definitive answers on the RTS rates and times are difficult to make based on these data, they remain illustrative and represent the best available evidence at this moment. In the future, another interesting research direction involves tracking elite athletes’ mental health directly from injury through treatment and rehabilitation. This enables early detection of mental health symptoms, allowing for timely intervention and providing the right support and necessary help.

## Conclusion

Different treatment options for OLAs allowed for an RTS rate of 96% at any level, a return to preinjury level of 72%, and an RTP rate of 64%. Clinical outcomes showed good results. The results of the present study can be used in the decision-making process between the physician and the elite athlete to inform them about the expected RTS rates and times for the different treatment options for OLAs. Furthermore, directly monitoring the mental health of elite athletes from the moment of injury to the end of the treatment and rehabilitation process is a promising area for research. This strategy facilitates the prompt identification of mental health symptoms, empowering physicians to intervene quickly and provide the required assistance and support.
